# Strain in shock-loaded skeletal muscle and the time scale of muscular wobbling mass dynamics

**DOI:** 10.1038/s41598-017-13630-7

**Published:** 2017-10-16

**Authors:** Kasper B. Christensen, Michael Günther, Syn Schmitt, Tobias Siebert

**Affiliations:** 1 Motion and Exercise Science, University of Stuttgart, Allmandring 28, 70569 Stuttgart, Germany; 2 Biomechanics and Biorobotics, Stuttgart Centre for Simulation Sciences (SC SimTech), University of Stuttgart, Allmandring 28, 70569 Stuttgart, Germany; 3 Institute of Sports Science, Friedrich–Schiller–University, Seidelstraße 20, 07749 Jena, Germany

## Abstract

In terrestrial locomotion, muscles undergo damped oscillations in response to limb impacts with the ground. Muscles are also actuators that generate mechanical power to allow locomotion. The corresponding elementary contractile process is the work stroke of an actin-myosin cross-bridge, which may be forcibly detached by superposed oscillations. By experimentally emulating rat leg impacts, we found that full activity and non-fatigue must meet to possibly prevent forcible cross-bridge detachment. Because submaximal muscle force represents the ordinary locomotor condition, our results show that forcible, eccentric cross-bridge detachment is a common, physiological process even during isometric muscle contractions. We also calculated the stiffnesses of the whole muscle-tendon complex and the fibre material separately, as well as Young’s modulus of the latter: 1.8 MPa and 0.75 MPa for fresh, fully active and passive fibres, respectively. Our inferred Young’s modulus of the tendon-aponeurosis complex suggests that stiffness in series to the fibre material is determined by the elastic properties of the aponeurosis region, rather than the tendon material. Knowing these stiffnesses and the muscle mass, the complex’ eigenfrequency for responses to impacts can be quantified, as well as the size-dependency of this time scale of muscular wobbling mass dynamics.

## Introduction

A common working condition of skeletal muscles during animal locomotion is active contraction during acceleration through space. In particular, if an animal’s locomotion requires repulsion from a solid substrate like soil or wood with the distal ends of the limbs contacting the substrate surface at finite impact velocities, shock-wave-like accelerations are induced to the bones. These shock waves are transmitted to the limb muscles^1–3^ via their suspensions and contact areas to the bones and adjacent muscles. Accordingly, shock waves that propagate through activated muscle tissue interfere with the asynchronous work of many myosin heads which build cross-bridges^4^ between myosin and actin filaments within the sarcomeres. It can be expected that a variety of compressive, shear, and torsional wave modes occur.

Since the earliest days of modern, quantitative muscle *ex-vivo*-experiments in the seventeenth century^5^, there have been no examinations, to the best of our knowledge, on the mechanics of muscular contraction in accelerated, non-steady-state conditions in which shock waves propagate through active fibres due to their inertia interacting with visco-elasticity. Accordingly, there is no knowledge about the mechanical characteristics of cross-bridges loaded during this fundamentally physiological impact situation so far.

**Figure 1:**
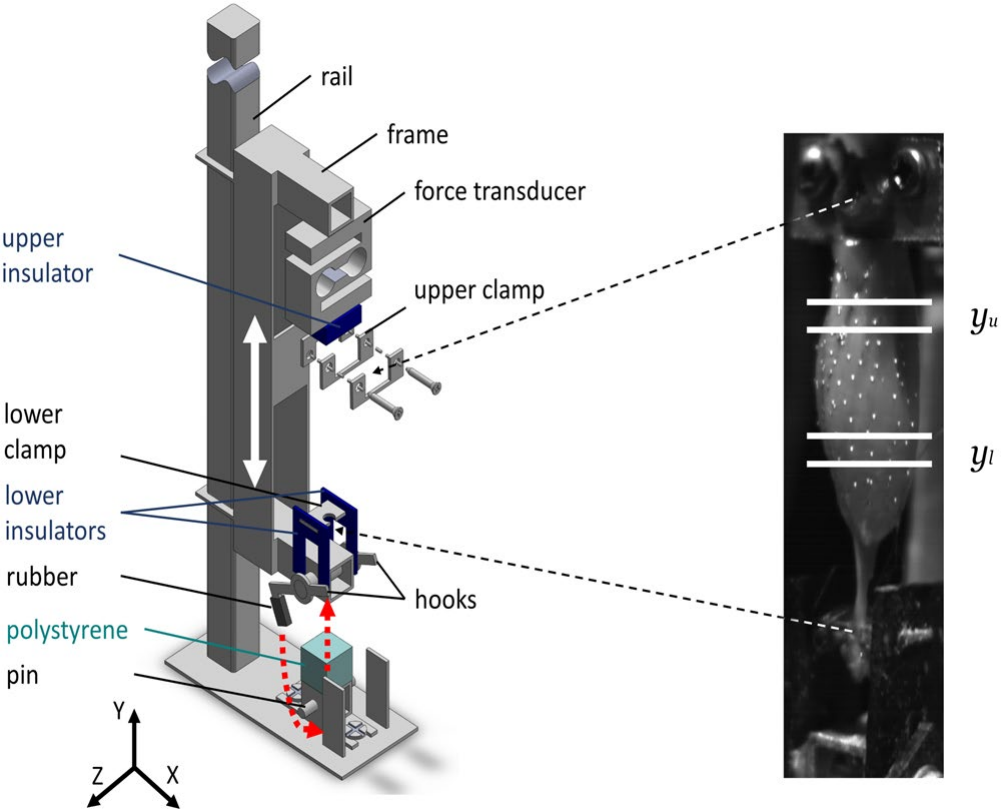
Experimental setup for impact experiments of GAS muscle-tendon complex (MTC). (**A**) Technical drawing of the C-shaped frame, which was freely movable (symbolised by the white double arrow) in the direction along the rail (see MM: ‘*Experimental setup*’). (**B**) Photo of a MTC specimen fixed in the frame directly before TD. The white spots are spherical steel markers with diameters of 0.4 mm adhering to the belly surface. Due to falling velocity (ca. 0.8 ms^−1^) and exposure time (0.5 ms), markers appear as smeared. Marker arrays between horizontal, white lines determining *y*_*u*_ and *y*_*l*_ were used for strain calculation (see MM: ‘*Detecting touch-down, calculating accelerations and strain*’).

In this situation, however, wave propagation is a typical response phenomenon of condensed matter, that is observable by time-varying strain. To examine whether cross-bridges in active skeletal muscles may be forcibly detached when strained by shock waves in the physiological range, we dropped nine specimens (*N* = 9) of isolated rat (*Rattus norvegicus*, Wistar) muscle (*m. gastrocnemius medialis* and *lateralis*: GAS) that was clamped *ex vivo* into a C-shaped frame (Fig. 1A) on the ground. For detailed information, see ‘Materials and Methods’ (MM): ‘*Data acquisition, marker tracking, and digital filtering’* and *‘Muscle preparation*’, respectively. The muscle-tendon complex (MTC) was fixed to the frame with small bone tissue pieces of the calcaneus and femur left at the end of the distal and proximal tendons, respectively, and subsequently stretched to its optimal length, which was inferred from literature^6^. To measure local strain ${\epsilon }_{CE}$ of the fibre material part of the MTC, labelled as ‘contractile element’ (CE), we patterned the muscle belly surface with 50 to 60 high-grade steel markers, with 0.4 mm diameters, and captured their kinematics by high-speed cameras at 1825 Hz sampling frequency during a fall and impact response. The ground was covered by polystyrene, and chosen drop height provided a vertical frame velocity of ca. 0.8 m s^−1^. With this, impact intensity and duration were in the physiological range^7–9^. We restricted the experimental condition to muscles that were oriented vertically, and we solely analysed strains in the longitudinal fibre direction.

## Results and Discussion

### Muscle strain and fatigue

We calculated the fibre material strain ${\epsilon }_{CE}={\rm{\Delta }}{L}_{CE}/{L}_{CE\mathrm{,0}}$ from a length *L*_*CE*_ spanning the fibre material in the centre of the muscle belly and a corresponding reference length *L*_*CE*,0_: Δ*L*_*CE*_ = *L*_*CE*_ − *L*_*CE*,0_. The reference length *L*_*CE*,0_ = *y*_*u*_ − *y*_*l*_ was the distance between the mean vertical positions of an upper (*y*_*u*_) and a lower (*y*_*l*_) marker subarray. Each subarray contained four to eight markers (Fig. 1B). The reference length (*L*_*CE*,0_ = 18.2 ± 0.8 mm) was the trial-specific *L*_*CE*_ at frame touch-down (TD; see MM: ‘*Detecting touch-down, calculating accelerations and strain*’).

**Figure 2:**
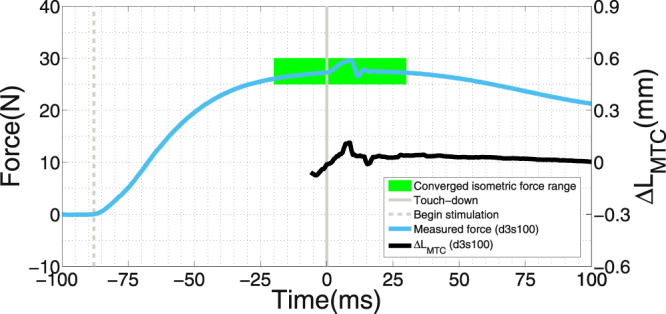
Example of experimental data: MTC force and MTC length around touch-down. Light blue, thick line: force transducer signal (left ordinate). Black, thick line: MTC length excursion Δ*L*_*MTC*_ (right ordinate) from MTC length *L*_*MTC*,0_ at TD. Vertical, dashed line: start of stimulation at *t* = −87 ms. Vertical, grey line: touch-down (TD) at *t* = 0. The green, shaded area indicates the time period in which the isometric force *F* was determined as the maximum low-frequency value from the force transducer trace, that is, with additionally excepting the (high-frequency) impact period from TD to 15 ms later. Usually, *F* was the value to which the force trace converged in the 10–15 ms after the impact period. The force decreased thereafter, which was a reproducible characteristic phenomenon in our experiments, particularly in the least fatigued muscles. In some experiments, the maximum in the force trace, except for the impact peak, was reached before TD. If force at TD and isometric force *F* differed by more than 5%, the trial was excluded from analysis (see MM: ‘*Exclusion criteria*’).

All dropping trials were done with fully active muscles, except for the last trial, which was performed with the stimulation switched off (passive).The muscle force was measured by a force transducer serving as a rigid connector between the suspending clamp of the upper tendon and the frame. Six to eleven trials per isolated muscle specimen were carried out as described in greater detail below. Figure 2 displays an example of the time courses of the force transducer signal and the calculated MTC length change. With our current spatial resolution, no strong and reliable statements about strains more local than an average for *L*_*CE*,0_ ≈ 1.8 cm in the centre of the belly can be made. A preliminary step to reduce *L*_*CE*,0_ values by shifting the lower subarray up in one exemplary trial resulted in ca. *L*_*CE*,0_ ≈ 1.2 cm. As a result, the peak of the strain signal was further delayed by ca. 1–2 ms. The strain amplitude was reduced by ca. 30%, which is a first indication of a wave pattern.

**Figure 3:**
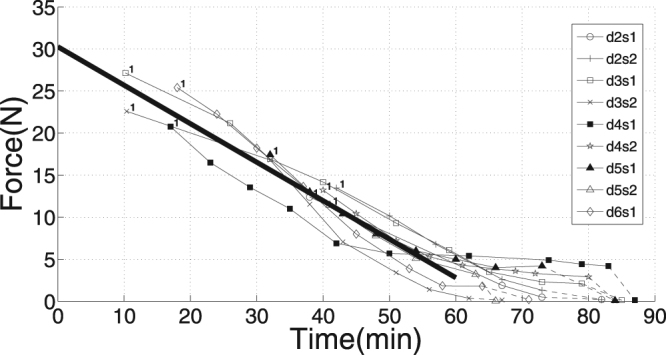
Initial (isometric) muscle force *F* at TD versus time after muscle extraction (*N* = 9). Only trials with a TD force of at least 95% of its isometric value were included, where the latter was defined by the value to which the force transducer signal converged shortly before or after TD (see Fig. 2). Trials were named (see key top right) after consecutive days of experiments (‘d#’), the first (‘s1’) or second (‘s2’) specimen extracted that day, and the trial number (starting with first as ‘00’). The thick, solid line is a linear fitting line to all data points below 60 min. The extrapolation to the instant of extraction at 0 min was assumed to represent the maximum isometric force *F* = *F*_*max*_ = 30 N. In Figs 4–7, the number of trials included is lower due to further exclusion criteria (see MM: ‘*Exclusion criteria*’).

Our experimental conditions correspond to total ischaemia, a condition in which force decline has been described as fatigue^10–12^. Each muscle specimen fatigued with consecutive trials, which can be seen from the trial-specific isometric force *F* decreasing almost linearly with time after muscle extraction (Fig. 3). Therefore, we indicate in the remainder of this paper increasing fatigue with decreasing isometric force *F*. Isometric force saturation tendencies were seen after 30–45 min at levels between 2% and 13% of maximum (non-fatigued) isometric force (*F*_*max*_). By linear extrapolation from 60 min back to the instant of extraction (thick, solid line in Fig. 3), we estimated a mean maximum value of *F* = *F*_*max*_ = 30 N, which can be expected from our anatomy (Table 1) and literature data (see Appendix ‘*Isometric force from physiological cross-sectional area (PCSA)*’, in particular, maximum mammalian muscle stress values).

**Table 1:** Anatomical data given as the mean value ± standard deviation of the nine specimens (*N* = 9).

**Description**	**Symbol**	**Data**	**Unit**	**Source**
Animal mass	${ {\mathcal M} }_{animal}$	413 ± 16	g	measured
GAS mass	${\mathcal M}$	2.0 ± 0.3	g	measured
MTC length at 90°	*L*_*MTC*,90°_	43 ± 0.3	mm	measured
MTC length in frame	*L*_*MTC*,0_	45^*^	mm	*L*_*MTC*,90°_ + 2
Belly length	*L*_*belly*,0_	33^†^	mm	*L*_*MTC*,0_ − *L*_*tendon,0*_
Reference length	*L*_*CE*,0_	18 ± 0.8	mm	measured
Proximal tendon length	*L*_*prox*,0_	2^*^	mm	literature
Distal tendon length	*L*_*dist*,0_	10 ± 0.35	mm	measured
Total tendon length	*L*_*tendon*,0_	12^*^	mm	*L*_*prox*,0_ + *Ldist*,0
Maximum belly ACSA	*A*_*CE*,0,*max*_	109 ± 4.5	mm^2^	measured
Minimum belly ACSA	*A*_*CE*,0,*min*_	73 ± 3.6	mm^2^	measured
Tendon ACSA	*A*_*tendon*,0_	2.8 ± 0.2	mm^2^	measured

We calculated the anatomical cross-sectional area (ACSA) by approximating the geometrical form of the area with a half-ellipse. One half-axis was measured as half the width of the muscle projection in the frontal view (Fig. 1B) right before TD. The second half-axis was calculated as the width along the frontal visual axis; this number was calculated from the shot of another camera at the same instant, and this camera was positioned to view along an axis rotated by 40° against the frontal view axis (partial side view).

^*^Our measured values of MTC length at 90° ankle and knee angles (*L*_*MTC*,90°_) and of distal tendon length (*L*_*dist*,0_) as well as the very short proximal tendon length (*L*_*prox*,0_) are equal (within 1 mm) to those published elsewhere^6^.

^†^ *L*_*belly*,0_ = 32 mm measured by others^47^.

Our isometric force decline lies within the range of other ischaemic studies. In these studies, force decline ranges from 95% of the isometric force in cat *m. soleus* and 90% in *m. gastrocnemius medialis and lateralis* both after ca. 23 minutes of ischaemia^13^ to 58% isometric force decline after 1 hour in rabbit *m. anterior tibialis* ^14^. Despite these declines, reperfusion restored 100% of isometric force in cat *m. soleus* and *m. gastrocnemius* ^13^ (‘… the soleus fully recovers in about 5 min while the recovery of the gastrocnemius takes 10–15 times longer.’), and 87% in rabbit *m. tibialis anterior* ^14^. Further studies^15,16^ stated full recovery (>90%) after 1 hour of ischaemia, proving that rapid fatigue within 1 hour is a metabolic adaptation to limited oxygen, nutrient, ion, and hormone availability, and also impeded waste removal, rather than any cellular process permanently being disabled by necrosis.

### Calculating muscle eigenfrequency and scaling

**Figure 4:**
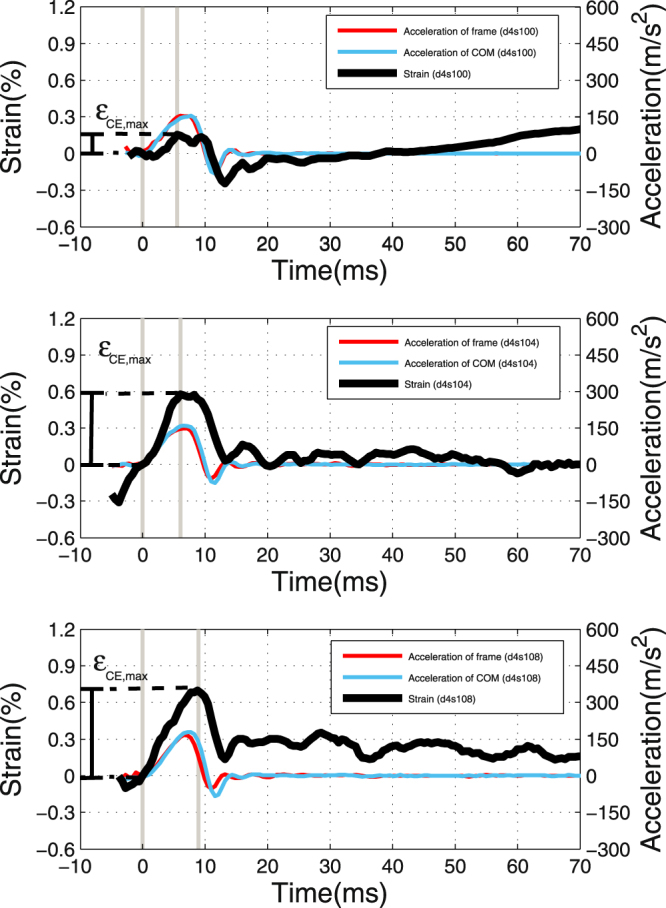
Time courses of CE strain and accelerations of COM and frame. Plots of the local fibre material strain ${\epsilon }_{CE}$ (black, thick, solid line: ${\epsilon }_{CE}\,=\,0$ at TD), net vertical MTC acceleration *a*_*COM*_ (light blue, thin, solid line: arithmetic mean of all steel markers’ values approximating the centre of mass, i.e., COM kinematics), and vertical acceleration of the suspending frame *a*_*frame*_ (dark red, thin, solid line) for three trials (from top to down) of the same muscle specimen: ‘d4s100’, ‘d4s104’, and ‘d4s108’ (naming: Fig. 3). The vertical, grey lines indicate time instants of TD (*t* = 0 s) and the maximum dynamic strain amplitude ${\epsilon }_{CE,max}$ during shock response (*t* > 0 s).

**Figure 5:**
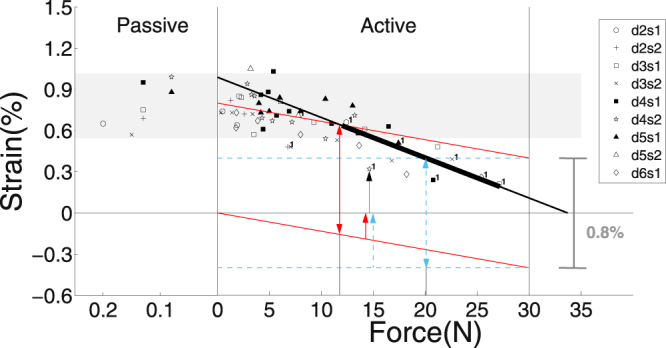
Maximum shock-wave-induced (‘dynamic’) fibre material strain ${\epsilon }_{CE,max}$ after TD versus isometric force *F* at TD (*N* = 9). Data for fully active muscles on the right side of *F* = 0 and for non-stimulated (‘passive’) muscles on the left. The grey-shaded region indicates a range of values from trials with passive muscle. Horizontal, blue, dashed as well as sloped, red, solid baselines at ${\epsilon }_{CE,max}$ < 0 symbolise initial strain hypothesised for cases (i) and (ii), respectively (see Appendix ‘*The initial and dynamic strain contributions*’). Dynamic strain values at which cross-bridges may be forcibly detached in cases (i) and (ii) are symbolised by corresponding lines at ${\epsilon }_{CE,max}$ > 0, where both are placed at ${\rm{\Delta }}{\epsilon }_{CE,max}$ = 0.8% distances from their respective baselines. Red, solid and blue, dashed arrows from ${\epsilon }_{CE,max}$ < 0 to ${\epsilon }_{CE,max}$ = 0 at *F* ≈ 15 N indicate initial strain values for cases (i) and (ii), respectively. The black arrow from ${\epsilon }_{CE,max}$ = 0 to the first trial of specimen ‘d4s2’ is one example of measured dynamic strain. The predicted force limits for forcible cross-bridge detachment are 20.1 N (blue, dashed double arrow) and 11.8 N (red, solid double arrow) for cases (i) and (ii), respectively. The black, thick, solid line represents the linear fitting line for ${\epsilon }_{CE,max}$ using all data points at *F* > 12 N–the thin extensions extrapolate to *F* = 0 and *F* = *F*_*max*_ = 30 N.

The maximum dynamic strain (in excess of the initial strain, see Appendix ‘*The initial and dynamic strain contributions*’) in response to frame impact was reached at 10.4 ± 2.5 ms after TD, which was on average 2.7 ms delayed to the instant of maximum vertical acceleration of the MTC’s centre of mass (COM: position as arithmetic mean of all steel marker positions) and 0.9 ms before the COM acceleration returning to zero (Fig. 4). The maximum dynamic strain (examples in Fig. 4) increased with muscle fatigue (Fig. 5), that is, with initial force *F* decreasing down to ca. a third of *F*_*max*_ (*F* = 10 N) and saturation occurring at the level of passive muscle for lower forces. The muscle including its tendon parts was stiff enough for its COM to closely follow the frame kinematics with a delay that slightly increased with time (see MM: *‘Detecting touch-down, calculating accelerations and strain’*).

**Figure 6:**
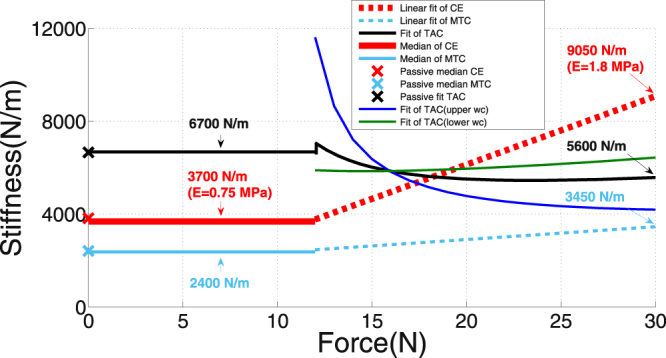
Linear interpolations ${\mathscr{K}}=a\cdot F+b$ versus isometric force *F* at TD in the region *F* > 12 N for stiffness values of CE $({{\mathscr{K}}}_{CE})$, MTC $({{\mathscr{K}}}_{MTC})$, and TAC $({{\mathscr{K}}}_{TAC})$. ${{\mathscr{K}}}_{CE}$ and ${{\mathscr{K}}}_{MTC}$ were measured (Fig. S3), whereas ${{\mathscr{K}}}_{TAC}$ was inferred from Eq. (8) and Eq. (7), respectively. Horizontal lines represent the respective median values for trials with active muscles in the region *F* < 12 N. Crosses represent the respective median values for all trials with passive muscles (for more details see Appendices ‘*Isometric force from physiological cross-sectional area (PCSA)*’, ‘*Calculating dynamic muscle force change and the elements’ stiffnesses*’, and ‘*Young’s moduli–a comparison to literature*’). The dark blue and green lines illustrate the uncertainty of TAC stiffness inferred on the basis of the linear fits (Eq. (12)) to MTC and CE stiffnesses (Fig. S3). The combined effects of MTC and CE stiffness dependencies on force are assumed to both follow the lower slopes in their worst case examination in Fig. S3 and result in the dark green line for the TAC stiffness dependency. Vice versa, combining both MTC and CE worst cases with upper slopes gives the dark blue line for the TAC stiffness dependency.

The circular eigenfrequency of a MTC exposed to a sudden stretch or force change (as in the impact situation) can be predicted as

1 $$\omega =\sqrt{\frac{{{\mathscr{K}}}_{MTC}}{ {\mathcal M} }}$$

directly from muscle mass ${\mathcal M}$ ≈ 2 g and MTC stiffness ${{\mathscr{K}}}_{MTC}$ (Fig. 6). Accordingly, the eigenfrequency values $f=\frac{\omega }{2\pi }$ are 209 Hz (${{\mathscr{K}}}_{MTC}$ ≈ 3450 N m^−1^) and 174 Hz (${{\mathscr{K}}}_{MTC}$ ≈ 2400 N m^−1^) for fully active and passive muscle, respectively. These eigenfrequency values explain why MTC dynamics is strongly bound to frame kinematics (COM acceleration delayed by 0.6 ms within impact duration, see MM: ‘*Detecting touch-down, calculating accelerations and strain*’). First, the oscillation period (ca. 5 ms: acceleration signals in Fig. 4) as the inverse of the eigenfrequency must be compared to twice the impact duration (ca. 20 ms). Second, the subtle feature of the delay increasing with time indicates viscous frame-MTC forces that dynamically stiffen elastic coupling. Independent of the strength of the frame-MTC coupling, the impact duration is mainly determined by the polystyrene cushion stiffness and frame mass, because the latter (ca. 52 g) is much higher than muscle mass ${\mathcal M}$.

Equating the frame to leg bone kinematics, the impact duration of ca. 10–15 ms is practically the same in the hindlimbs of running rats^17^, comparably small mammals^7^, and humans^3^. Peak values of COM acceleration are also similar for a running rat’s GAS (${a}_{COM,max}$ = 165 m s^−2^ see MM: ‘*Detecting touch-down, calculating accelerations and strain*’) and running human’s leg muscles^3^ (*a*_*shank*,*max*_ ≈ 320 m s^−2^, *a*_*thigh*,*max*_ ≈ 160 m s^−2^). Yet, the phase relations of the leg’s wobbling^18,19^ masses in response to the impact clearly differ. In humans, maximum vertical accelerations of the segmental muscle masses occur ca. 5 ms (shank) and 20 ms (thigh), after maximum leg bone acceleration^3,20^. During impacts, wobbling mass dynamics thus have a higher functional relevance in bigger animals because there is an increasing temporal separation of bone and muscular movement. Furthermore, one can expect that the MTC mass

2 $$ {\mathcal M} =\alpha \cdot \rho \cdot {L}^{3}$$

scales like body mass with a characteristic length *L*, with the proportionality factor *α*, and the mass density *ρ*. Accordingly, muscle stiffness

3 $${{\mathscr{K}}}_{MTC}=E\cdot \frac{A}{L}=\beta \cdot E\cdot L$$

scales linearly with *L* due to the cross-sectional area scaling as

4 $$A=\beta \cdot {L}^{2},$$

with *β* being a second proportionality factor and *E* Young’s modulus. With this, the circular eigenfrequency (Eq. (1)) scales as

5 $$\omega ={L}^{-1}\cdot \sqrt{\frac{E}{\rho }\cdot \frac{\beta }{\alpha }}={ {\mathcal M} }^{-\frac{1}{3}}\cdot \sqrt{E\cdot \beta \cdot {(\alpha \cdot \rho )}^{-\frac{1}{3}}}$$

with length or mass^21^, respectively. Using Eq. (5) and assuming that MTC mass roughly scales with body mass, we can predict a characteristic value of muscular wobbling mass eigenfrequency in humans of *f*_*human*_ = (0.4 kg/70 kg${)}^{\frac{1}{3}}$ ⋅ *f*_*rat*_ ≈ $\frac{1}{5.6}$ ⋅ 200 Hz ≈ 35 Hz. Experimentally, the range 25–40 Hz has been found for human shank and thigh muscles^3^ [Figs 3–6].

### CE strain and regional stiffness in response to impact

To support our argument of measuring strain as an indicator of forcible cross-bridge detachment, we shortly review the basic idea behind force generation by a tilting myosin head within a cross-bridge. Mechanically, during the force-generating process (i.e. the work stroke) the myosin head must be strongly bound to actin. The term ‘strongly bound’ implies limited adherence forces at the attachment site, and an analogue of a myosin head would be a suction cup that adheres to an actin active site, where the adherence force is limited by the material properties of both adherents. As there are head-internal and filament-internal contributions to elasticity, this elementary work stroke process and the functional existence of fibre-internal stiffness are, mechanically, inseparably connected. Thus, more or less by definition of the term ‘strongly bound’, any other potential binding state, which may be suggested by observation and postulated by models of muscular contraction, is ‘weakly bound’ in a mechanical sense. Weakly bound states can thus not contribute to fibre-internal stiffness as an insignificant force in the location of attachment would resist strain. However, while energy dissipation will always be induced by forcible detachment of strongly bound states, such dissipation may also occur for weakly bound states. Such energy loss has also been termed ‘protein friction’^22–27^.

After an event of forcible detachment, we would expect that a previously strongly bound cross-bridge undergoes ‘repriming’^28,29^, which may be the re-attachment at an active site on actin that would be located at one, two, or more active sites further from the site of detachment. Such repriming occurs within about 5–8 ms in response to shortening^28^. In response to 5 nm lengthening steps per cross-bridge^29^, which corresponds to ca. 0.5% dynamic strain, many cross-bridges seem to remain attached as can be seen from the initial force response, which is a peak that is due to elastic distortion. Repriming after lengthening seems to be slower than during shortening. It will thus not occur during shock-wave-induced impacts lasting about 10 ms (see MM: ‘*Detecting touch-down, calculating accelerations and strain*’). If cross-bridges are forcibly detached during shock waves, we would therefore expect (a) energy dissipation by protein friction, (b) rapid relaxation of all non-detached cross-bridges^29,30^ on the time scale of a few milliseconds after a force peak Δ*F*, (c) repriming of cross-bridges after fibre lengthening on the time scale of ca. 20 ms^29^, and (d) re-attachment dynamics of the assembly of cross-bridges on an even longer time scale (ca. 40 ms in isometric conditions^31^) with reduced forces in response to the intermittent phenomena of force increase Δ*F* and relaxation decrease.

**Figure 7:**
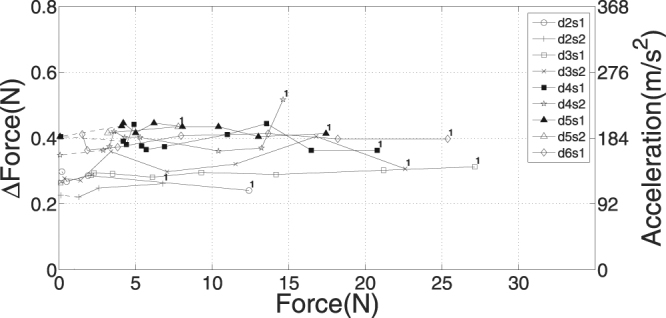
Muscle force peak (force difference Δ*F*) during shock-wave-induced (‘dynamic’) fibre material strain after TD versus isometric force *F* at TD (*N* = 9). The force difference Δ*F* was calculated as the corresponding vertical acceleration *a*_*COM*_ of the MTC’s COM (right ordinate) plus gravitational acceleration multiplied by muscle mass ${\mathcal M}$ (see Appendix ‘*Calculating dynamic muscle force change and the elements’ stiffnesses*’). The COM position was calculated as the arithmetic mean of all steel marker positions.

The measured strain increases with fatigue down to ca. *F* = 10 N (Fig. 5), and the measured dynamic force increases Δ*F* are practically independent of fatigue (Fig. 7). If the decrease in isometric force *F* seen during fatigue was due to a decrease in force per cross-bridge with the number of cross-bridges remaining the same, the CE stiffness should remain constant with fatigue because the stiffness of a single cross-bridge is practically constant down to ca. 10% *F*_*max*_ in initially isometric conditions^30,32^ and down to ca. 50% *F*_*max*_ in isotonic contractions^33^. Alternatively, if the force per cross-bridge remained constant with fatigue, but the number of cross-bridges decreased, the CE stiffness should decrease along with fatigue. According to Fig. 6, CE stiffness ${{\mathscr{K}}}_{CE}$ decreases from 9050 Nm^−1^ in fresh muscle to 3700 Nm^−1^ at *F* = 12 N. This is a strong indication that the CE stiffness decrease is due to a decreasing number of cross-bridges at *F* > 12 N.

Measured MTC stiffness ${{\mathscr{K}}}_{MTC}$ (2400–3450 Nm^−1^), calculated CE’s Young’s modulus *E*_*CE*_ (0.75–1.8 MPa), and inferred stiffness ${{\mathscr{K}}}_{TAC}$ (5,600–6,700 Nm^−1^) of the ‘tendon-aponeurosis complex’ (TAC: arranged in series to the CE) matched well with values from literature (see Appendix ‘*Young’s moduli–a comparison to literature*’). From this, it follows that ${{\mathscr{K}}}_{TAC}$ is dominated by properties of the aponeurosis region rather than the tendon because the values are about 50 times lower than would be expected from mammalian tendons’ Young’s modulus *E*_*tendon*_ ≈ 1.5 GPa^34,35^ and our rats’ anatomical data (Table 1).

Shock waves induced dynamic strains of ca. 0.2% in the least fatigued case (Fig. 5). The net strain of all fibre-internal serial elasticities in the actin and myosin filaments and the cross-bridges at maximum isometric force is ca. 0.4%^30,32,36^ in the non-fatigued case. Forces to forcibly detach one myosin head from actin have been measured to scatter around 9 pN^37^, whereas estimations of the maximum isometric force of a cross-bridge range from 2 pN^30^ to more modern, higher values^33^, and up to more than 10 pN^32^. Moderate values of ca. 4 pN come from energetic estimations^31,38^. Mechanical, structural, and energetic approaches seem to converge at about 5 pN^39^. Additionally, force saturation in eccentric contractions, as another possible measure for a limit of forcibly detaching myosin heads, which indicates muscle ‘giving’, has been quantified to be 1.4–2.0 times the isometric force^40–43^.

Altogether, we extract the three following assumptions used for the line of reasoning in the remainder of this paragraph: (a) other possible sources of forces that act in parallel to a cross-bridge and may resist stretching (e.g., titin^44,45^) are neglected, (b) also an initial strain of 0.4% in the serial elasticities is assumed at maximum isometric force *F* = *F*_*max*_, and (c) roughly double this force value is needed to forcibly detach a cross-bridge, corresponding to 0.8% limit strain. If we further assume in a model case (i) that fibre-internal stiffness ${{\mathscr{K}}}_{CE}$ is located solely in the myosin heads (horizontal, blue, dashed lines in Fig. 5), the initial plus shock-wave-induced, dynamic strain according to our experimental data would allow cross-bridges to stay bound down to 20.1 N (67% of *F*_*max*_: vertical, blue, dashed double arrow in Fig. 5). In the more realistic model case (ii) of ${{\mathscr{K}}}_{CE}$ distributed across heads, actin, and myosin (vertical, red, solid lines in Fig. 5), cross-bridges are predicted to stay bound down to 11.8 N (40% of *F*_*max*_: vertical, red, solid double arrow in Fig. 5). This is practically the very same force boundary value *F* = 12 N (40% of *F*_*max*_) that was found in the stiffness analysis (Fig. 6), where the changes of CE and MTC stiffness characteristics with force *F* saturate at constant levels of the passive muscle. Specifically, we found that CE and MTC stiffnesses decreased from 9050 N m^−1^ to 3700 N m^−1^ and 3450 N m^−1^ to 2400 N m^−1^, respectively, for a force decrease from 100% to about 40% of *F*_*max*_ (Fig. 6). For a discussion of the local joint force *F* see Appendix ‘*A reflection on muscle-internal mechanics during impact*’.

In our methodological approach, the muscle might be non-uniformly activated, in particular in the fatigued case. In addition, kinematics are measured solely on the surface. Thus, information about fibres within the muscle belly may be lacking or inaccurate. To gain direct evidence–beyond verifications addressed in Fig. 6–that the strain determined in our approach by signals from the muscle surface are also representative of the *in vivo* conditions of fibres within the muscle belly, for example, the use of high-speed X-ray cinematography would be promising.

Compared to single fibre preparations, additional mechanical properties become functional in macroscopic muscles. An essential property of an anatomically intact assembly of many muscle fibres (a macroscopic muscle) is the collective response of this assembly to an impact. In this, the mechanical coupling of the single fibres by passive, connective tissue within the assembly is inseparably incorporated into the wobbling mass response: A single fibre interacts with the inertia of all other muscle material in series and in parallel.

## Conclusion

In summary, the dynamic strain of rat GAS muscle fibres under physiological, shock-wave-induced stretch conditions saturates below a stress of about 40% of the maximum isometric value. As the fibres were initially at their optimum lengths, this saturation may be due to counteracting forces by passive, connective tissue within or surrounding the sarcomeres: initial, passive forces are ca. 0.1–0.2 N (abscissa of Fig. 5), which is comparable to the maximum dynamic force changes of 0.2–0.4 N (left ordinate of Fig. 7). Experiments at shorter initial fibre lengths will provide further insight. Our calculation of the wobbling mass eigenfrequency is already predictive regarding the overall muscle and therefore body size. It is, however, descriptive regarding an MTC’s physiological and anatomical design. A model that formulates the eigenfrequency as a function of the corresponding design parameters would thus allow to gain further insight into how functional demands under common shock wave conditions formed MTCs during evolution. However, we dare to state thus far that the frequency spectrum in terrestrial locomotion is broader in bigger animals: the ratio between the inverse of the wobbling mass eigenfrequency and the duration of bone impact dynamics is about six times higher in humans than in rats.

## Materials and Methods

### Ethics statement

All experiments were performed on rat (*Rattus norvegicus*, Wistar) muscles (*m. gastrocnemius medialis* and *lateralis*: GAS). Nine GAS specimens (*N* = 9) were extracted from freshly killed rats provided by another animal study that was approved according to Section 8 of the German animal protection law (Tierschutzgesetz, BGBl. I 1972, 1277). That study performed experiments on other leg muscles, and the rats had been anaesthetised with sodium pentobarbital (100 mg per 1 kg body mass). The applicants of the approved animal experiment study had no objection against GAS extraction immediately after the rats’ death. The results of their experiments were not impaired by GAS extraction. Anatomical data specified as the mean of the nine specimens can be seen in Table 1.

### Data acquisition, marker tracking, and digital filtering

To study the shock-wave-induced kinematics of rat (*Rattus norvegicus*, Wistar) muscles (*m. gastrocnemius medialis* and *lateralis*: GAS) *ex vivo* (see MM: ‘*Muscle preparation*’), the frontal area of the GAS was patterned by pressing the muscle belly on a prepared array of high-grade steel markers (spheres, nominal diameter 0.4 mm, mensuration N0, IHSD-Klarmann, 96047 Bamberg, Germany) as seen in Fig. 1B. The GAS was then fixed in an aluminium frame (Fig. 1A) which was dropped on the ground (see MM: ‘*Experimental setup*’). Local muscle kinematics was recorded with four high-speed cameras, each recording 256 × 1,024 pixels per sample at 1825 Hz sampling rate (HCC-1000 BGE, VDS Vosskühler, 07646 Stadtroda, Germany). All cameras were equipped with a 25 mm focal length lens (Xenon 25/0.95, Schneider-Kreuznach, 55543 Bad Kreuznach, Germany) and custom-made 2 mm extension tubes for minimising the focusing distance. With this, the width of one pixel was equal to ca. 0.1 mm. To counter the inverse relation between recording frequency and exposure time of a camera’s sensor array, sufficient light conditions were provided by two stroboscopes (MultiLED PT, GSvitec GmbH, 63571 Gelnhausen, Germany) that flashed asynchronously at a rate of 20 kHz. The cameras were placed along a semicircle with radius of ca. 15 cm on the open side of the C-shaped frame, and all were focused on the frontal surface of the belly located in the semicircle’s centre. The imaging planes were aligned in parallel to the vertical (rail) axis. Two-dimensional images from a particular camera were respectively calibrated, including distortion correction in linear proportion to distance to the image centre. After automatic marker tracking using ‘DigitizingTools’ (Hedrick Lab, University of North Carolina, Chapel Hill, USA; coded in MATLAB, The MathWorks, Natick, USA), the marker positions were digitally filtered using a moving average with a symmetric window of five samples. Accelerations were calculated with a symmetrical, first order (two point) central difference formula.

### Muscle preparation

The MTC of the GAS was freed from its surrounding tissues with the exception of small bone tissue pieces of the calcaneus and femur for fixating the MTC in the frame. The muscle was stimulated (Aurora Scientic 701C) with 500 *μ*s square wave pulses of 10 V (three times the twitch threshold) at 100 Hz to ensure tetanic contraction during the trials, as recommended by a previous study^46^. Experiments were conducted with the GAS contracting isometrically at optimal fibre length while falling. The series of falling experiments with each specimen were finalised by a trial without stimulation, i.e., with passive muscle fibres. To prevent desiccation, the GAS surface was moisturised once between trials with Ringer’s solution of 38 °C temperature from a small spray flask. The experiments were conducted at room temperature (23–25 °C).

### Experim**e**ntal setup

To emulate *in vivo* impact conditions, impact surface stiffness and falling height of the frame were adjusted to reproduce the kinematics of a rat’s distal hindlimb after TD when running, assuming that a paw’s TD velocity normal to the ground is ca. 1 m s^−1^ ^7–9^. The right-angled, C-shaped aluminium frame had an upper and lower clamp construction for MTC fixation between its cantilever arms (Fig. 1A). The backbone of the frame was 120 mm long, and its two arms protruded by 40 mm. The total mass of the frame including the force transducer was 52 g. This transducer (KD24S 20N, ME-Meßsysteme GmbH, 16761 Henningsdorf, Germany) was positioned above the upper clamp and was insulated from the latter by a plastic cuboid (Fig. 1A, all insulators are shown in dark blue). The lower clamp was an aluminium hook jig connected to the frame via two shiftable, inverted-U-shaped plastic insulators that allowed MTC length adjustment. For MTC fixation, the femur piece was placed between the two upper, U-shaped clamps, which were then screwed to their third inverted, U-shaped counterpart. At the MTC’s opposite end, the calcaneus bone piece was placed in the hook jig of the lower clamp.

A rectangular aluminium profile, serving as a rail for the frame, was fixed on the base plate of the whole experimental device. After being released with an electromagnet, the frame fell freely, but guided (white double arrow for indication) by the rail, and was eventually decelerated by compressing a polystyrene cuboid with a mass of 0.041 ± 0.0001 g and a surface area of 1 cm^2^. Both the electromagnet and the polystyrene were in vertical alignment with the overall frame-muscle centre of mass. To ensure minimal rebound and oscillation of the frame after TD, two hooks at the lower arm of the frame were locked in place under the horizontal pin at maximum polystyrene compression (mechanism indicated in Fig. 1A by red, dashed, curved, and straight arrows). To minimise jerk, the hook tips were covered with a rubber layer.

The conductance of tendon material is poor. In order to properly stimulate the muscle during fall, impact, *and* shock response, we wired the positive electrode to the upper frame clamp (very short tendon) and wrapped the negative electrode around the lower clamp. The bent wire termination was put into blunt contact with the dorsal CE part between lateral and medial *m. gastrocnemius* at the distal fibre-tendon junction, where the electrode was held in place by muscle tissue adhesiveness. Since the falling duration Δ*t*_*fall*_ of the frame was constant across trials (fixed falling height) whereas the time Δ*t*_*F*_ to converge to the isometric force *F* (plateau region in Fig. 2) after the start of stimulation varied (depending on the degree of fatigue), the electromagnet, the force transducer, and the cameras were all triggered by an adjustable hardware that generated a delay to stimulation onset, which corresponded to the difference between Δ*t*_*fall*_ and Δ*t*_*F*_. The latter was 95 ms in the trial with the freshest specimen (*F* = 27 N). From there, Δ*t*_*F*_ decreased linearly by ca. 2.4 ms N^−1^ to finally saturate below *F* = 8 N, resulting in an approximately 49% decrease in muscle conductivity velocity, which is similar to other ischaemic results (38%)^13^. For the relation between isometric force *F* and time after muscle extraction see Fig. 3.

### Detecting touch-down, calculating accelerations and strain

Touch-down (TD) was the point in time when the frame made contact with the polystyrene. It was determined in each trial as the point before the earliest instant at which the second time derivative of the COM position (acceleration) had raised above noise level. As no delay between the acceleration signals of frame and COM was detectable in our experiments around TD, either signal could be used in principle to determine TD. However, detecting TD with the COM acceleration was favourable because this method proved more reliable than using frame marker acceleration. Although allowing an extended template size was possible for frame marker tracking (using ‘WINanalyze’, Mikromak Service, Berlin, Germany), and this setup could achieve a spatial noise of just 0.1 pixel for this frame marker, using an array of belly markers (individual noise: 1 pixel) yielded a slightly better signal-to-noise ratio for the array than for the frame marker.

The instants at which maximum accelerations occurred were 7.4 ± 1.0 ms and 7.7 ± 0.9 ms for the frame and COM, respectively. While the COM signal lagged the frame signal by 0.3 ms, on average, a t-test showed that this lag was insignificant at *p* > 0.05. The maximum COM acceleration values ${a}_{COM,max}$ were 165 ± 23 m s^−2^ (see right ordinate Fig. 7), which were reached, on average, 2.7 ms earlier than maximum strain. However, strain kinematics are much more variable (see standard deviation of 2.5 ms): in some trials, maximum strain was even reached before maximum COM acceleration, particularly in non-fatigued muscle with low strain maxima (Fig. 4, top). The impact duration measured as the time spent from TD to frame (or bone) acceleration returning to zero was 10.7 ± 0.9 ms. Zero COM acceleration occurred at 11.3 ± 0.7 ms, and the delay to zero frame acceleration was doubled to 0.6 ms as compared to instants of their maxima, which was significant on a level *p* < 0.05 using a t-test.

To determine belly strain ${\epsilon }_{CE}$, an upper and lower range of each ~10% of total muscle length was identified. The vertical placement of both marker subarrays were nearly symmetrically positioned around the midpoint of the belly (location of maximum cross-sectional area *A*_*CE*,0,*max*_), as seen in Fig. 1B. The horizontal, white lines across the belly represent the subarray limits and thereby confine what we denote as the ‘contractile element’ (CE) in this study. The representative vertical position of each marker subarray (*y*_*u*_ and *y*_*l*_ with *u* for ‘upper’ and *l* ‘lower’) was calculated as the arithmetic mean of the vertical positions of all markers in this subarray. The reference length *L*_*CE*,0_ that defined zero percent strain is the distance between the vertical subarray positions ${L}_{CE\mathrm{,0}}=|{y}_{u}-{y}_{l}|$ measured at TD in each trial (*L*_*CE*,0_ = 18 ± 0.8 mm). In Fig. 1B, the aponeuroses extend on both lateral sides of GAS, i.e., the field of view. Thus, by solely using markers from the centre of the *y*_*u*_ and *y*_*l*_ regions, care was taken to analyse the kinematics of fibres alone rather than any aponeurosis material.

### Exclusion criteria

Not all data were suitable for use within our analyses; therefore, exclusion criteria at different stages of the data processing were used. In particular, we excluded the following: (I) an entire trial if the force at TD was less than 95% of the trial-specific isometric force, where the latter was determined as the force value to which the force transducer signal converged shortly before or after the impact response (see also Fig. 2); (II) an entire trial if material shortening $({\epsilon }_{CE}\, < \,0)$ preceded material elongation as an initial response to TD (e.g., Fig. S1, top); (III) a marker in a trial if it had obviously glided across the muscle surface during the experiment; and (IV) a marker in a trial if it showed phase and/or amplitude irregularities (either in coordinate position or acceleration) when compared to all other markers. Note that all data analysed and presented in Figs 4–7 did not meet the exclusion criteria (I)–(IV). As an exception, data regarding the initial force, which are shown in Fig. 3, only needed to pass criterion (I).

## Electronic supplementary material

Supplementary Information
