# Supplementary material for: Strain in shock-loaded skeletal muscle and the time scale of muscular wobbling mass dynamics
Source: Sci Rep. 2017 Oct 16;7:13266. doi: 10.1038/s41598-017-13630-7 (PMC5643554; doi:10.1038/s41598-017-13630-7)
Supplement: Supplementary file 1 — Supplementary Information [file 41598_2017_13630_MOESM1_ESM.pdf]

Supplementary Information Appendix

Strain in shock-loaded skeletal muscle and the time scale of muscular wobbling mass dynamics

Kasper B. Christensen*, Michael Günther, Syn Schmitt, Tobias Siebert

*Corresponding author. E-mail: kasper.christensen@inspo.uni-stuttgart.de

This PDF file includes:

Appendix:

Isometric force from physiological cross-sectional area (PCSA)
The initial and dynamic strain contributions
Calculating dynamic muscle force change and the elements' stiffnesses
Young's moduli – a comparison to literature
A reflection on muscle-internal mechanics during impact
Figs. S1 to S3
References [48–66]

Appendix

Isometric force from physiological cross-sectional area (PCSA)

The maximum isometric muscle tension σ_{max} is between $2.25 \cdot 10^5\,\mathrm{Nm}^{-2}$ [53] and $3 \cdot 10^5\,\mathrm{Nm}^{-2}$ [54]. If PCSA $P_{CE,0}$ and the pennation angle α are known, the maximum isometric muscle force F_{max} can be estimated. We found $P_{CE,0}$ and α values in two cases [55,56]. In case (a) [55], $P_{CE,0}$ and α were measured for the *gastrocnemius medialis* and *lateralis* muscles separately. The *lateralis* muscle accounted for 56% of the GAS PCSA ($P_{CE,0} = 111\,\mathrm{mm}^2$) and $\alpha = 14°$ was found in both muscles. In case (b) [56], measurements were made for only *m. gastrocnemius medialis* ($54\,\mathrm{mm}^2$, $\alpha = 21°$). Assuming a 44% contribution of this muscle, which is in line with Eng et al. [55], the overall GAS PCSA in the work of Kadhiresan et al. [56] would then have been $P_{CE,0} = 123\,\mathrm{mm}^2$. Due to the same boundary condition defining $L_{MTC,90°}$ in case (i) [55], we also assumed $\alpha = 14°$ for our specimens and calculated our mean PCSA ($P_{CE,0} = 112\,\mathrm{mm}^2$) as

$$P_{CE,0} = \frac{A_{CE,0,max}}{\cos\alpha} \tag{6}$$

Based on the σ_{max} range given above, maximum isometric force can be calculated as $F_{max} = \sigma_{max} \cdot A_{CE,0} = \sigma_{max} \cdot P_{CE,0} \cdot \cos\alpha$, which would yield values between 24.2 N and 32.3 N in case (i) and between 25.8 N and 34.5 N in case (ii). Compared to these estimations, we found $F_{max} = 30\,\mathrm{N}$ by extrapolation (Fig. 3).

The initial and dynamic strain contributions

Fibre material strain can be divided into two contributions: 'initial strain' due to the initial, isometric force and additional 'dynamic strain' due to the impact response. We measured the latter, and three examples are plotted in Fig. 4. For the initial strain, two limit cases of locating elasticity as a potential cause are conceivable: strain is either (i) solely located in the myosin heads or (ii) distributed across all sarcomere structures that act in series, that is, heads and actin plus myosin filament backbones. By further assuming in the case (i) that decreasing initial force was solely due to the reduced number of attached cross-bridges, the initial strain of the fibre material would always the same, regardless of the force – that is, any single cross-bridge generates its maximum force (about 0.4% [30,35,48,49,50], see bottom, horizontal, blue, dashed line in Fig. 5). In any other case, the most simple model relating initial fibre force to initial strain would be a linear stiffness (see bottom, sloped, red, solid line in Fig. 5 with zero strain at zero force and -0.4% at maximum isometric force), which is our case (ii). The overall maximum fibre strain during impact can then be calculated by the sum of the estimated initial strain and the measured dynamic strain. For one trial (no. 1 of 'd4s2'), initial and dynamic strain contributions are visualised in Fig. 5 by vertical arrows – a blue, dashed arrow (i) and a red, solid arrow (ii) for the two initial strain cases as well as a black, solid arrow on top of them for the measured dynamic strain. The sloped, thick, solid line represents the

best linear fit of measured dynamic strains in all trials at initial forces higher than 12 N; the thinner linear line represents extrapolations to zero force and zero strain. This force boundary value at 12 N comes from a stiffness analysis of the MTC (Fig. 6).

Calculating dynamic muscle force change and the elements' stiffnesses

Beyond describing CE-internal kinematics by strain ϵ, we used Newton's second law $\Delta F = \mathcal{M} \cdot (a_{COM} + g)$ to calculate the dynamic force difference ΔF between MTC ends in response to the impact. The symbols are: muscle mass $\mathcal{M}$, COM acceleration a_{COM}, and gravitational acceleration g. Because CE mass is much higher than TAC mass, ΔF also well approximates the force difference between CE ends. On average, maximum values of ΔF are 0.36 ± 0.07 N, and they are practically independent of fatigue (see left ordinate in Fig. 7). Since the dynamic force difference ($\Delta F(t)$) as well as COM deflection relative to the frame $\Delta L_{MTC}(t) = \big(y_{COM}(t) - y_{frame}(t)\big) - (y_{COM}(t = 0) - y_{frame}(t = 0))$ and CE-internal elongation $\Delta L_{CE}(t)$ are all known as functions of time t in each single trial, the MTC ($\mathcal{K}_{MTC}$) and CE ($\mathcal{K}_{CE}$) stiffnesses can be calculated based on all data points in the respective force-elongation relations $\Delta F(\Delta L_i(t))$ between TD and the instant of $a_{COM,max}$, with $i = MTC$ (examples in Fig. S2) or $i = CE$.

From this, the stiffness

$$\mathcal{K}_{TAC} = \frac{\mathcal{K}_{CE} \cdot \mathcal{K}_{MTC}}{\mathcal{K}_{CE} - \mathcal{K}_{MTC}} \tag{7}$$

of the 'tendon-aponeurosis complex' (TAC) was inferred based on the model idea that the MTC consists of a mass that is suspended to the frame (or bone) by two compliant elements arranged in series (CE and TAC) given the overall MTC and the local CE stiffness values.

We examined whether our calculated CE and MTC stiffness values may depend monotonically on isometric force F by using the simplest approach, that is, we fit the data with a linear function. The corresponding straight-line fits through all data points above 12 N are plotted in Fig. S3. We extracted this force boundary value at 12 N by separating the calculated $\mathcal{K}_{MTC}(F)$ and $\mathcal{K}_{CE}(F)$ data points into two ranges of approximately constant values (low forces) and assumed linear change with force F (high forces: straight lines), and searching for the boundary value between them with the best continuity of the inferred $\mathcal{K}_{TAC}(F)$ curve (see black, solid line). Except for the cases where either (i) the slopes of $\mathcal{K}_{MTC}(F)$ and $\mathcal{K}_{CE}(F)$ are zero or (ii) both stiffness values have their x-intercept at zero force, the $\mathcal{K}_{TAC}(F)$ curve will be a non-linear function. The inferred curve represents an almost constant value around $\mathcal{K}_{TAC}(F) \approx 5{,}600\,\mathrm{N\,m^{-1}}$ down to ca. $F \approx 18$ N, where the $\mathcal{K}_{CE}(F)$ curve starts to undergo the $\mathcal{K}_{TAC}(F)$ curve. Non-linear deviation of inferred $\mathcal{K}_{TAC}(F)$ from near-constancy increases with a further decrease in force down

to the boundary $F = 12\,\mathrm{N}$. Yet, three points should be noted: (i) $\mathcal{K}_{TAC}(F)$ curvature may be a technical artefact of the fact that $\mathcal{K}_{CE}(F)$ and $\mathcal{K}_{MTC}(F)$ themselves do not exactly trend linearly with physiological reality; (ii) the independent inferences from below and above $F = 12\,\mathrm{N}$ are almost the same at the boundary itself, namely, $\mathcal{K}_{TAC}(F) \approx 6{,}700\,\mathrm{N\,m^{-1}}$; (iii) the uncertainty of the values $\mathcal{K}_{TAC}(F) = 5{,}600\text{-}6{,}700\,\mathrm{N\,m^{-1}}$ is the same as in other experiments on solely the *m. gastrocnemius medialis* head [42], and the predicted absolute values are slightly lower than found in two other analyses based on Hill-type models [6,42]. Here, $\mathcal{K}_{TAC}$ is equivalent to $\mathcal{K}_{SEC} = 9000 \pm 1{,}400\,\mathrm{N\,m^{-1}}$ (pers. comm. to T. Siebert) [42] and $\mathcal{K}_{SEC} = 8{,}000\,\mathrm{N\,m^{-1}}$ [6] there.

Young's moduli – a comparison to literature

Young's modulus $E_i = \mathcal{K}_i \cdot \frac{L_{i,0}}{A_{i,0}}$ is defined as a local material property that can be found from known macroscopic properties of a finite mass portion i, including stiffness $\mathcal{K}_i$, reference length $L_{i,0}$, and cross-sectional area $A_{i,0}$. With the chosen reference length $L_{CE,0} = 0.0182\,\mathrm{m}$ (Table 1) in the centre of the muscle belly, $A_{CE,0} = 1.09 \cdot 10^{-4}\,\mathrm{m^2}$ the maximum estimate of the anatomical cross-sectional area (ACSA) within $L_{CE,0}$ (Table 1), and $\mathcal{K}_{CE} \approx 9050\,\mathrm{N\,m^{-1}}$ the stiffness of fully active, fresh muscle fibres, we calculated the lower limit of their Young's modulus to be $E_{CE} \approx 1.5\,\mathrm{MPa}$. Due to low pennation angles $\alpha \approx 20^\circ$ at optimal fibre length [55,56,47], our ACSA and physiological cross-sectional area (PCSA) values found in literature [55,56] are practically identical. For fixed $A_{i,0}$ and $L_{i,0}$, Young's modulus scales linearly with $\mathcal{K}_i$. Thus, in strongly fatigued and passive muscle fibres with $\mathcal{K}_{CE} \approx 3700\,\mathrm{N\,m^{-1}}$, we found $E_{CE} \approx 0.6\,\mathrm{MPa}$ as a minimum, which is ca. 40% of the stiffness and modulus values for fresh, fully active fibres. Due to using an upper limit for ACSA, these values of Young's modulus represent lower limits. Using $A_{CE,0} = 0.73 \cdot 10^{-4}\,\mathrm{m^2}$ estimated at its upper (proximal) end, which is the minimum ACSA value within $L_{CE,0}$, we estimated an upper limit of Young's modulus of $E_{CE} \approx 2.3\,\mathrm{MPa}$ for fresh and $E_{CE} \approx 0.9\,\mathrm{MPa}$ for fatigued/passive muscle fibres. Assuming volume constancy, homogeneity, and uniformity for fibres and the belly's [5,57,58,59,52], Young's modulus equals the shear modulus. With this, our results match others' data [60] well; this is demonstrated by the local shear values that were found at the Z-disc and M-line locations: 2.6 MPa and 1.3 MPa, respectively, in a fully active fibre as well as 1.7 MPa and 1.2 MPa, respectively, in a passive fibre.

TAC stiffness $\mathcal{K}_{TAC}$ according to Eq. (7) is calculated (inferred) from

$$\mathcal{K}_{MTC} = (\mathcal{K}_{CE} \cdot \mathcal{K}_{TAC}) / (\mathcal{K}_{CE} + \mathcal{K}_{TAC}) \quad . \tag{8}$$

$\mathcal{K}_{TAC}$ contains all compliances in series to the fibre material, and is equivalent to what is often referred to as 'serial elastic element' (SEE) or 'serial elastic component' (SEC) in muscle modelling. The basic notion is described in three papers [61,62,63]. TAC stiffness is dominated by aponeurosis rather than tendon properties in rat GAS. The line of reasoning is concise. First, to check whether

TAC stiffness was dominated by tendon properties, we calculated the tendon stiffness

$$\mathcal{K}_{tendon} = E_{tendon} \cdot \frac{A_{tendon,0}}{L_{tendon,0}} \tag{9}$$

from anatomical data $L_{tendon,0} = 0.012\,\mathrm{m}$, $A_{tendon,0} = 2.8{\cdot}10^{-6}\,\mathrm{m}^2$, and a well-established literature value for Young's modulus of mammalian tendon material $E_{tendon} = 1.5\,\mathrm{GPa}$ [33, 34, 64, 65, 66] as $\mathcal{K}_{tendon} = 350\,\mathrm{kN\,m}^{-1}$. This $\mathcal{K}_{tendon}$ value would be about 50 times higher than our upper limit of inferred TAC stiffness values $\mathcal{K}_{TAC} = 6{,}700\,\mathrm{N\,m}^{-1}$. Conversely, an upper limit estimate of TAC's Young's modulus

$$E_{TAC} = \mathcal{K}_{TAC} \cdot \frac{L_{TAC,0}}{A_{TAC,0}} \tag{10}$$

can be calculated from assuming (a) the highest stiffness value $\mathcal{K}_{TAC} = 6{,}700\,\mathrm{N\,m}^{-1}$ inferred from our analysis, (b) the smallest cross-sectional area value $A_{TAC,0} = A_{tendon,0} = 2.8{\cdot}10^{-6}\,\mathrm{m}^2$ measured for rat GAS tendon, and (c) the length $L_{TAC,0} = L_{MTC,0} - L_{CE,0} = 0.027\,\mathrm{m}$ that bridges the distance between the fibre material (analysed within $L_{CE,0}$) and the frame. The corresponding theoretical, upper limit $E_{TAC} = 64\,\mathrm{MPa}$ is 23 times lower than mammalian tendons' Young's modulus. Alternatively, the lower limit $E_{TAC} = 2.1\,\mathrm{MPa}$ can be calculated from using (a) the lowest TAC stiffness value $\mathcal{K}_{TAC} = 5{,}600\,\mathrm{N\,m}^{-1}$ occurring in our analysis and (b) the minimum fibre ACSA value $A_{TAC,0} = A_{CE,0} = 0.73{\cdot}10^{-4}\,\mathrm{m}^2$ at the boundary between fibre material (CE) and TAC.

As $\mathcal{K}_{CE}$ results from the serial arrangement of all local elasticities within the cross-sectional areas along the finite length $L_{CE,0}$, the most likely value of inferred E_{CE} should be approximately the arithmetic mean value of the upper and lower limits at each force, that is, $E_{CE} = 1.9\,\mathrm{MPa}$ for fresh and $E_{CE} = 0.75\,\mathrm{MPa}$ for fatigued/passive muscle. The local net material property E_{TAC} in a belly's aponeurosis region may well increase continuously – when approaching the aponeurosis-tendon junction – from $E_{TAC} = E_{CE} = 0.75 \ldots 1.9\,\mathrm{MPa}$ at the fibre-aponeurosis boundary to $E_{TAC} = E_{tendon} = 1.5\,\mathrm{GPa}$ at the aponeurosis-tendon junction. Besides the fibre activity, this continuous change will depend on the geometrical arrangement of the aponeurosis and the fibres as well as on the shifting fibre-to-aponeurosis ratios of material contributions within the belly and on the belly surface. Average values of TAC's Young's modulus in the range $E_{TAC} = 2.1\text{-}64\,\mathrm{MPa}$ supports this idea.

A reflection on muscle-internal mechanics during impact

In the subsection '*Calculating dynamic muscle force change and the elements' stiffnesses*', we explained how we used Newton's second law to calculate – from the kinematics of the centre of the distributed muscle masses (COM) estimated by marker tracking – the dynamic force difference ΔF between muscle ends (origin and insertion) that must have acted to accelerate the COM. As additional information, we measured the initial, isometric force F with a force sensor in series to the muscle (plotted in Fig. 3 as a function of time after muscle

extraction). If the muscle masses are suspended to a rigid construction (i.e., the frame), one can calculate a finite distance between the COM and the suspension positions that approximates an anatomical length, namely, the muscle-tendon complex (MTC) length. Our mechanical analysis of a real muscle implies at least two further prerequisites: (i) we wanted to formulate a mechanical model of the MTC to potentially subdivide it into further sub-elements, and (ii) the MTC is suspended to a single rigid construction at both ends. Both prerequisites are implied in our calculation of the MTC length, and (ii) means that it does not matter whether MTC length changes are calculated with respect to the upper or lower suspension point.

Our initial idea was that the minimum mechanical model description starts with just one net suspending structure for the COM: a spring acting between COM and one suspension point; note that any length change calculated this way represents the length change of all MTC material. Consistent with this, the calculated force difference ΔF, which is proportional to the COM acceleration, is interpreted as the net force change that acts on the MTC spring while it changes its length by ΔL_{MTC}. Likewise, a net MTC stiffness can be calculated. We further know that (iii) few percent of the MTC masses are located in the tendons, which justifies estimating COM acceleration from kinematic information of fibre material solely. We then can use this to make a first MTC length subdivision within the model, namely, into the length of mass distribution itself (termed CE) and the remaining material in series. Thus, the COM of the (masses in the) CE are accelerated in space as calculated above from ΔF, and we determine an additional length change ΔL_{CE} of this CE part superposed to the COM movement. This CE length change between the upper and lower ends of the CE region subtracted from all MTC length change is the length change ΔL_{TAC} of all MTC parts in series to the CE region, which we indicate by TAC.

Using these arguments, we can now explain in more detail our model idea of two deforming (length changing) structures in series that suspend the muscle mass to the skeleton or frame, respectively: while the CE can locally change length in any instant due to finite fibre stiffness, which can easily induce, e.g., double fibre stress (and thus mean force F in a cross-sectional area) by doubling strain as compared to initial, isometric strain, the force difference ΔF between both ends of the MTC (and thus CE) provides its acceleration in space. Note that we measured reliable, static (isometric) force values only before touchdown. ΔF should also be reflected by the sum of both length changes in the upper and lower anatomical structures in series to CE, that is, the upper and lower TAC parts, respectively.

In a nutshell, F and ΔF are, for the time being, two independent mechanical variables that are probably locally and continuously distributed during wave propagation. The variable F represents the concept of 'joint force' that causes linear momentum transmission between two adjacent mass distributions (compare, e.g., the study [51] [appendix 2]) that can be calculated according to Newton's third law (action=reaction) in any conceived area of cutting through the muscle. The variable ΔF represents the difference in external forces that accelerates one selected mass distribution localised between its spatial boundaries

(‘joints’).

In a real muscle, the stress or force, respectively, and their gradients are expected to vary temporally and spatially during wave propagation. The local force F is the integral of stress over a finite cross-sectional area, and the force difference ΔF is an integral of a force gradient distribution over a finite distance along the gradient. Adding the idea of elastic cross-bridges, changes in strain ϵ and force F are proportionally connected, which applies to both temporal and spatial variations – that is, rates and gradients, respectively – provided there is no forcible detachment. For example, a local gradient in strain generates a local force gradient and thus finite force differences ΔF for finite distances along the gradients. As we could only spatially resolve the net (mean) strain in the whole fibre material region of ca. 18 mm using our present pioneering methodological approach, our strain signals (plotted in Figs. 4 and 5) represent the mean strain values along this CE region. With higher spatial resolution, local strain and thus stress or force F values, respectively, are probably distributed around their mean values.

References

48. Dobbie, I. *et al.* Elastic bending and active tilting of myosin heads during muscle contraction. *Nature* **396**, 383–387 (1998).

49. Linari, M. *et al.* The stiffness of skeletal muscle in isometric contraction and rigor: the fraction of myosin heads bound to actin. *Biophysical Journal* **74**, 2459–2473 (1998).

50. Decostre, V., Bianco, P., Lombardi, V. & Piazzesi, G. Effect of temperature on the working stroke of muscle myosin. *Proceedings of the National Academy of Sciences of the USA* **102**, 13927–13932 (2005).

51. Linari, S. W., Günther, M., Renjewski, D. & Seyfarth, A. Impulsive ankle push-off powers leg swing in human walking. *The Journal of Experimental Biology* **217**, 1218–1228 (2014).

52. McMahon, T. A. *Muscles, Reflexes, and Locomotion* (Princeton Univ. Press, Princeton, 1984).

53. Powell, P. L., Roy, R. R., Kanim, P., Bello, M. A. & Edgerton, V. R. Predictability of skeletal muscle tension from architectural determinations in guinea pig hindlimbs. *Journal of Applied Physiology* **57**, 1715–1721 (1984).

54. Reconditi, M. Recent improvements in small angle X-ray diffraction for the study of muscle physiology. *Reports on Progress in Physics* **69**, 2709–2759 (2006).

55. Eng, C. M. *et al.* Scaling of muscle architecture and fiber types in the rat hindlimb. *The Journal of Experimental Biology* **211**, 2336–2345 (2008).

56. Kadhiresan, V. A., Hassett, C. A. & Faulkner, J. A. Properties of single motor units in medial gastrocnemius muscles of adult and old rats. *The Journal of Physiology* **493**, 543–552 (1996).

57. Swammerdam, J. Earliest known experimental evidence for volume constancy of skeletal muscle during contraction [52]; first published by quotation not before 1669 (1663) [5].

58. Stensen (Stenonis), N. *Elementorum Myologiæ Specimen, seu Muscili Descriptio Geometrica* **2**, 61–111 (Stellae, Florence, 1667) [5].

59. Baskin, R. J., & Paolini, P. J. Volume change and pressure development in muscle during contraction. *American Journal of Physiology* **213**, 1025–1030 (1967).

60. Ogneva, I. V., Lebedev, D. V. & Shenkman, B. S. Transversal stiffness and Young's modulus of single fibers from rat soleus muscle probed by atomic force microscopy. *Biophysical Journal* **98**, 418–424 (2010).

61. Wilkie, D. R. The relation between force and velocity in human muscle. *The Journal of Physiology* **110**, 249–280 (1949).

62. Hill, A. V. The abrupt transition from rest to activity in muscle. *Proceedings of the Royal Society B* **136**, 399–420 (1949).

63. Hill, A. V. The series elastic component of muscle. *Proceedings of the Royal Society B* **137**, 273–280 (1950).

64. Ker, R. F. Dynamic tensile properties of the plantaris tendon of sheep (*Ovis aries*). *The Journal of Experimental Biology* **93**, 283–302 (1981).

65. Bennett, M. B., Ker, R. F., Dimery, N. J. & Alexander, R. M. Mechanical properties of various mammalian tendons. *Journal of Zoology* **209**, 537–548 (1986).

66. Pollock, C. M. & Shadwick, R. E. Relationship between body mass and biomechanical properties of limb tendons in adult mammals. *American Journal of Physiology* **266**, R1016–1021 (1994).

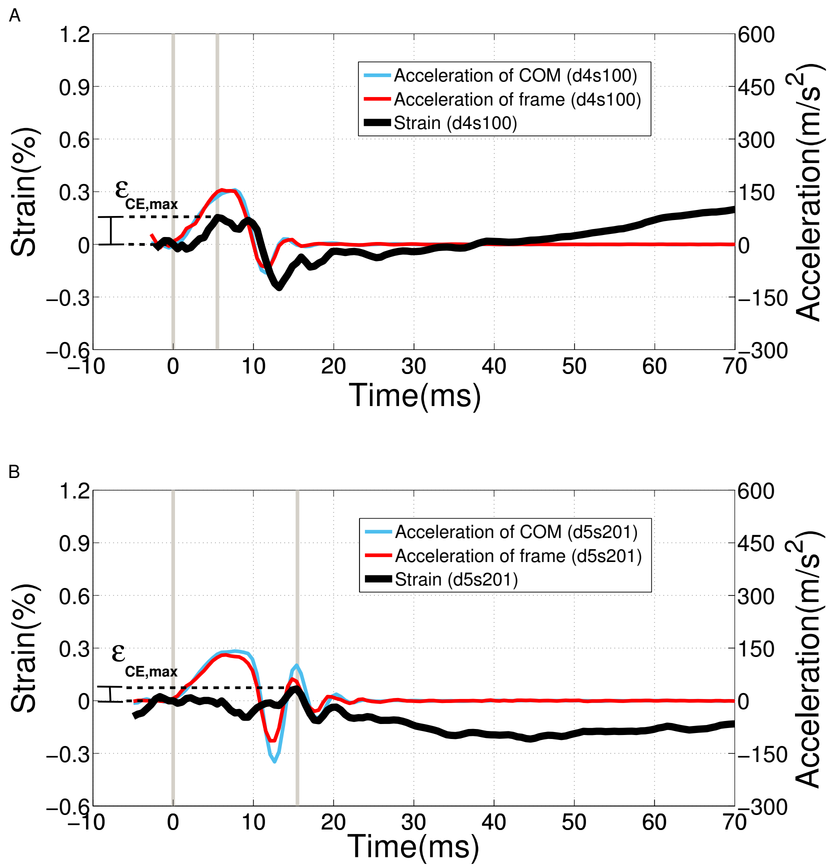

Fig. S1 | Time courses of CE strain and accelerations of COM and frame. Plots of the local fibre material strain ϵ_{CE} (black, thick, solid line: $\epsilon_{CE} = 0$ at TD), net vertical MTC acceleration a_{COM} (light blue, thin, solid line) – that is, the arithmetic mean of all steel markers' values approximating centre of mass (COM) kinematics – and vertical acceleration of the suspending frame a_{frame} (dark red, thin, solid line) for trials 'd4s100' (A) and 'd5s201' (B). Vertical, grey lines indicate time instants of TD ($t = 0$ s) and maximum dynamic strain amplitude $\epsilon_{CE,max}$ during shock response $t > 0$ s. The bottom trial 'd5s201' was *excluded* from our analysis because its earliest extreme in strain (at ca. 9 ms) was a *minimum* rather than a maximum as in the top trial 'd4s100' (at ca. 4.5 ms).

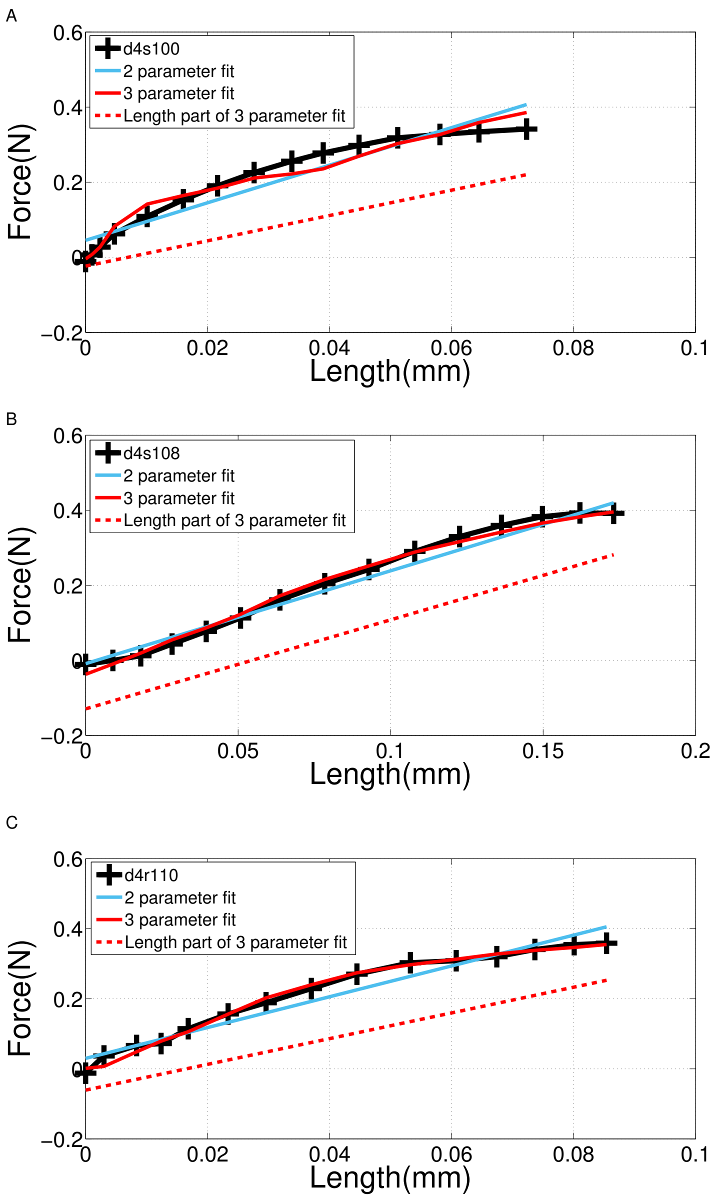

Fig. S2 | Force difference ΔF versus displacement ΔL_{MTC} of the MTC's COM in the initial phase (ca. 5 ms starting with TD) of shock-wave-induced strain. Panels from top to bottom show examples for three trials of the same muscle specimen 'd4s1': 'd4s100', 'd4s108', 'd4s110' (crosses). The force difference ΔF is calculated as the corresponding vertical acceleration a_{COM} of the MTC's COM (right ordinate in Fig. 7) plus gravitational acceleration and multiplied by the muscle mass $\mathcal{M}$. The COM position is calculated as the arithmetic mean of all steel marker positions on the muscle. L_{MTC} is the COM's vertical distance to a frame marker, and $L_{MTC,0}$ is this distance at TD. $\Delta L_{MTC} = L_{MTC} - L_{MTC,0}$ is the corresponding COM displacement. The functions ('3 parameter fit')

$$F(L_i(t)) = k \cdot L_i(t) + b + d \cdot \dot{L}_i(t) \qquad (11)$$

are also plotted using three parameters k, b, d to fit a linear function $F(L_i, \dot{L}_i)$ to measured data (here: $L_i = L_{MTC}$ and $F = \Delta F$). Accordingly, parameter k is a direct estimation of the MTC stiffness $\mathcal{K}_{MTC}$ (slope of dashed line) that results from the serial arrangement of muscle fibre (CE: $\mathcal{K}_{CE}$) and tendon/aponeurosis (TAC: $\mathcal{K}_{TAC}$) material. For comparison, the effect on estimating $\mathcal{K}_{MTC}$ when neglecting the damping parameter d is shown (slope of '2 parameter fit').

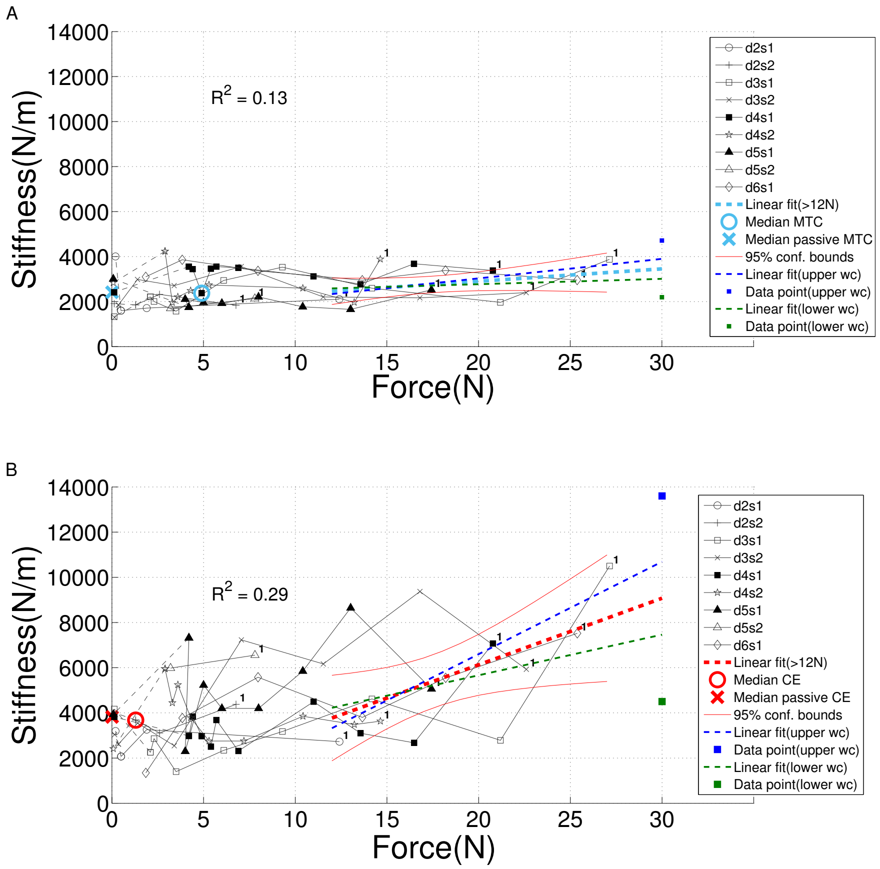

Fig. S3 | Measured stiffness values of MTC (top: $\mathcal{K}_{MTC}$) and CE (bottom: $\mathcal{K}_{CE}$) versus isometric force F at TD. For each single trial, these values were determined by fitting the linear relation Eq. (11) with three parameters (Fig. S2) to their measured force-length data and identifying slope parameter k as the respective element's stiffness. Through each of the two data clouds, a linear fit

$$\mathcal{K} = a \cdot F + b \qquad (12)$$

to all data points was calculated for $F > 12\,\mathrm{N}$. Additionally, the following are also plotted: (i) circles represent the median value for trials with active muscles in the region $F < 12\,\mathrm{N}$, and (ii) a cross represent the median value for all trials with passive muscles. To illustrate the uncertainty of the linear approximations of the stiffness dependencies on force (fatigue), the 'undisturbed linear fits' (light blue, thick, dashed line in A and red, thick, dashed line in B), we re-calculated the linear fits of Eq. (12) using two additional worst case (wc) data points that were hypothetically placed at the maximum observed force (plus and minus observed, maximum deviation from each undisturbed linear fit in A and B: blue and green squares, respectively). The corresponding linear fits, each including the respective wc data point, are plotted as dashed blue and green lines, respectively, in A and B. Additionally, the 95% confidence intervals for the undisturbed linear fits in A and B are plotted with red, thin, solid lines. Note that, although MTC stiffness does only weakly depend on force, the slope of the linear fit remains positive even in the wc of the hypothetically low stiffness value at maximum force.
